# Challenges in the diagnosis of fibrodysplasia ossificans progressiva with the *ACVR1* mutation (c.774G > C, p.R258S): a case report and review of literature

**DOI:** 10.1186/s13023-024-03363-y

**Published:** 2024-09-30

**Authors:** Siqi Yang, Rongrong Cui, Jialin Li, Ruchun Dai

**Affiliations:** National Clinical Research Center for Metabolic Diseases, Institute of Metabolism and Endocrinology, Central South University, Hunan Provincial Key Laboratory of Metabolic Bone Diseases, and Department of Metabolism and Endocrinology, The Second Xiangya Hospital of Central South University, 139 Middle Renmin Road, Changsha, Hunan 410011 China

**Keywords:** Fibrodysplasia ossificans progressiva, Mutation, Early diagnosis

## Abstract

The diagnosis of fibrodysplasia ossificans progressiva is missed or delayed because of its insidious precursors, especially in uncharacteristic cases. Fibrodysplasia ossificans progressiva, which mostly displayed the mutation c.617G > A, p.R206H, is characterized by congenital malformation of the great toe and progressive extra-skeletal ossification of ligaments, tendons and muscles. The mutation c.774G > C, p.R258S (HGVS: NC_000002.11:g.158626896 C > G) in activin A receptor type I is an infrequent etiology of fibrodysplasia ossificans progressiva and can present different clinical features. Awareness of these multiple clinical features will help endocrinologists in the early diagnosis of fibrodysplasia ossificans progressiva. We report a case of fibrodysplasia ossificans progressiva with the activin A receptor type I mutation c.774G > C, p.R258S, which was diagnosed before its ossifying period.

## Background

Fibrodysplasia ossificans progressiva (FOP) is an infrequent progressive genetic disease affecting one in a million individuals [1–3] and is featured by congenital great toe malformations and progressive extraskeletal ossification of ligaments, muscles and tendons. FOP occurs because of gain-of-function mutation in activin A receptor type I/activin-like kinase 2 (*ACVR1/ALK2*), and over 95% of the typical form of FOP patient population displayed the mutation c.617G > A, p.R206H. However, other mutations which cause FOP seemingly lead to clinical features that are different from the “classic”, which makes the diagnosis challenging. Patients with FOP are mostly born with the short, deviated, and monophalangic great toe, but there is no other symptom [4]. The first onset of FOP occurs at approximately 5 years of age [5]. Flare-ups are characterized by local inflammatory symptoms such as painful soft tissue swelling and loss of function, and heterotopic ossification (HO) will develop subsequently. The functional disability and limitations progress with age [6], and they ultimately die from cardiac and respiratory complications, such as pneumonia or right-sided heart failure. Analyzing genetic mutations in the *ACVR1/ALK2* gene by whole exome sequencing and Sanger sequencing is a reliable and safe method of FOP diagnosis. As yet, there is no effective established treatment for managing FOP. Reducing the risk of physical trauma and controlling inflammation are essential for all patients with FOP because tissue damage (falls, surgery, biopsies and intramuscular injections) and subsequent inflammation are the first step to trigger HO [7]. Corticosteroids, non-steroidal anti-inflammatory drugs (NSAID), bisphosphonates and rehabilitative care are used empirically in clinical practice to treat flare-ups, but there is no conclusive evidence. New effective medications are under development and some emerging targeted therapies for FOP are already researched in clinical trials.

The *ACVR1* gene (Ensembl: ENSG00000115170) is located on chromosome 2 consisting of 9 coding exons [8]. It codes a bone morphogenetic protein (BMP) type I receptor, known as ACVR1/ALK2 (UniProtKB: Q04771), which consists of 509 amino acids. The ACVR1 protein belongs to the transforming growth factor-β superfamily, which comprises an extramembrane N-terminal ligand-binding domain, a transmembrane (TM) domain, an intracellular glycine–serine-rich (GS) domain, and a protein kinase (PK) domain [9–10]. ACVR1 and the type II receptors BMPR2, activin receptor type-2 A, and activin receptor type-2B form hetero-tetrameric complexes [11]. The type II receptors transphosphorylate the GS domain of the type I receptors upon ligand binding, which results in activation of the PK domain of the type I receptor. Subsequently phosphorylating the small-mothers-against decapentaplegic (SMAD) 1/5/8 proteins involved in signal transduction [12]. In addition to canonical SMAD signaling, ACVR1 can also activate noncanonical signaling pathways [13]. In FOP patients, the mutant receptor enhances the phosphorylation of SMAD1/5/8, and upregulates the expression of target osteogenic genes, which induces endochondral differentiation [14].

We report the case of a male juvenile patient who carrieed a heterozygous mutation c.774G > C, p.R258S (HGVS: NC_000002.11:g.158626896 C > G) in *ACVR1* bringing about the replacement, R258S, in the kinase domain of the protein. He was finally diagnosed with FOP. On the basis of this case, we discuss unusual clinical signs and the early diagnosis of FOP to alert clinicians to pay more attention to this disease.

### Case report

A 15-year-old Chinese boy visited a local hospital with a chief complain of swelling in the scapular region for 10 days in April 2022. There was tenderness in the swelling district but the bilateral arms could be raised properly. Anti-inflammatory drugs was prescribed, and the swelling subsided in a few days. However, he developed waist pain when squatting, hip pain when raising legs, right axillary pain during passive traction, and suffered from difficulty in squatting because of pain in both knees. His neck was limited in forward flexion and backward leaning. The limitation of movement of the left shoulder was slight, but the upper arm could be lifted. The right shoulder joint was limited in abduction, lifting, and supination, and its elevation was restricted to a range of 60°. 3 months later, the patient was referred to the endocrinology department in our hospital.

**Fig. 1:**
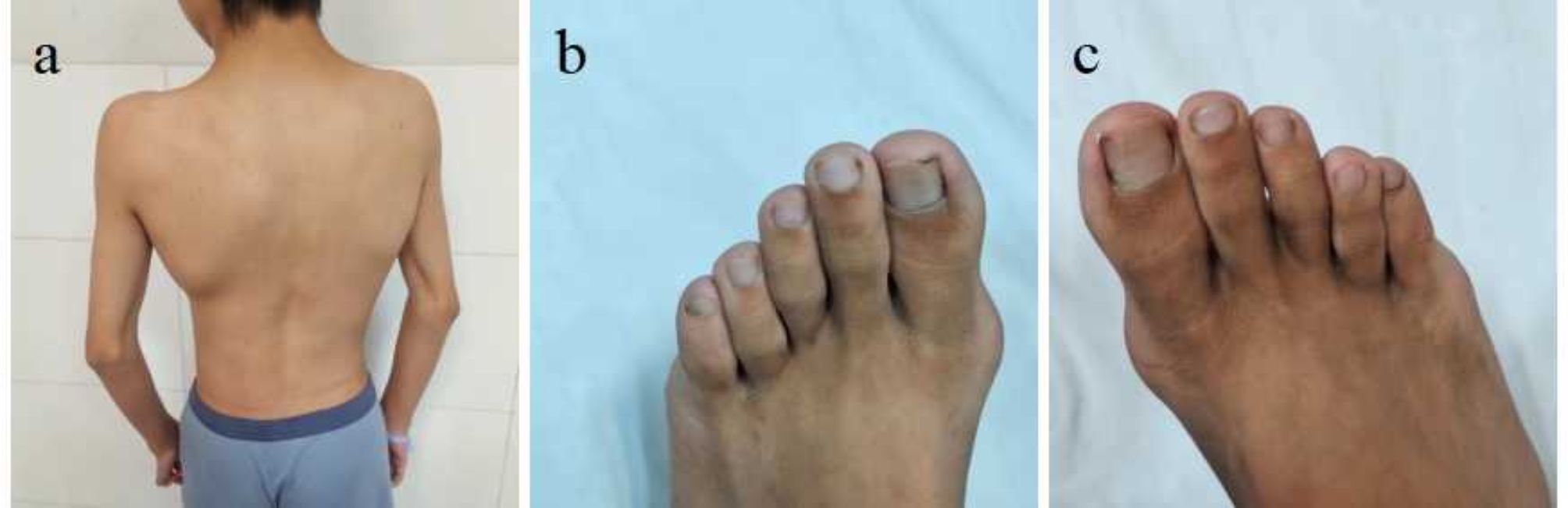
Clinical features of the patient. (**a**) Scoliosis; (**b**, **c**) Bilateral hallux valgus, short fourth toe

After admission, we found obvious scoliosis in this patient and both of his shoulders are tilted forward (Fig. 1a). In addition, he has mild and bilateral hallux valgus, and his right fourth toe was short (Fig. 1b, c). The patient had a thin body type, no blue sclera, and no cleft palate. He had limited internal rotation of both femurs and negative metacarpal signs bilaterally. He had no beard and his Adam’s apple is not obvious. His bilateral testes were approximately 6 cm in size. And he had no nocturnal penile tumescence or spermatorrhea. He belongs to nonconsanguineous Chinese parents. His family and birth history were non-contributory. Laboratory evaluation revealed that urinalysis, total blood count, kidney and liver function, the serum level of C reactive protein, erythrocyte sedimentation rate, inflammatory indicators, complements, immunity and autoimmunity profiles (Table 1) were all in their reference range, which helped us to rule out the possibility of rheumatoid arthritis. His parathyroid hormone level was 48.10 pg/ml (reference range, 18.50–88.00 pg/ml) and he had a 25-hydroxyvitamin D deficiency with a 25-hydroxyvitamin D level of 27 nmol/L (reference range, 75–250 nmol/L). And the patient had active bone turnover rate (Table 2). Some studies suggested the importance of whole exome sequencing as a precise, quick and economical method for identifying candidate gene and novel disease-causing variants in rare diseases [15–18]. Based on clinical signs and normal inflammatory indicators, a diagnosis of FOP was suspected, and we suggested he performed the genetic analysis with whole exome sequencing from a peripheral blood sample. Genomic DNA was isolated from peripheral blood according to the manufacturer’s instructions, and sheared into fragments of 200–500 bp in length. The ends were repaired and A tails were added, and then different barcodes of different specific sequences were ligated to both ends of the fragments to prepare DNA libraries. Sequences were captured using the MagPure Blood DNA TL Kit (Magen Biotechnology Co., LTD., Guangzhou, China), and the libraries were amplified by PCR and subjected to library quality tests by Qubit. After passing the test, high-throughput sequencing was performed using the AmCare Seq-2000 platform (Jiajian Biotechnology Co., LTD., Guangzhou, China). The sequencing results were analyzed by bioinformatics methods. Quality control was performed on the variant data, and variants with sequencing coverage depth below 20x were marked as low-quality variants. The quality control data of whole exome sequencing has been illustrated in Table 3. Variance analysis and interpretation was performed with pathogenic mutation database (ClinVar, HGMD, DECIPHER, ISCA, NCBI), normal population database (gnomAD, ExAC Browser, DGV), OMIM database and protein function prediction software (Polyphen-2, VEP, SIFT, REVEL). Single nucleotide polymorphisms and low-frequency benign variants were annotated. The pathogenicity of identified variants was analyzed according to the variant interpretation guidelines of American College of Medical Genetics and Genomics(ACMG) [19]. The identified variant was further validated by Sanger sequencing. The primer sequences for Sanger sequencing are as follows: F 5′‐GGTGTTTGAGAAAATTAAACAGCA‐3′, R 5′‐GGCTGCCTCCAAAATGACTA‐3′. The reference sequence NM_001105.5 of ACVR1 gene was used.

**Fig. 2:**
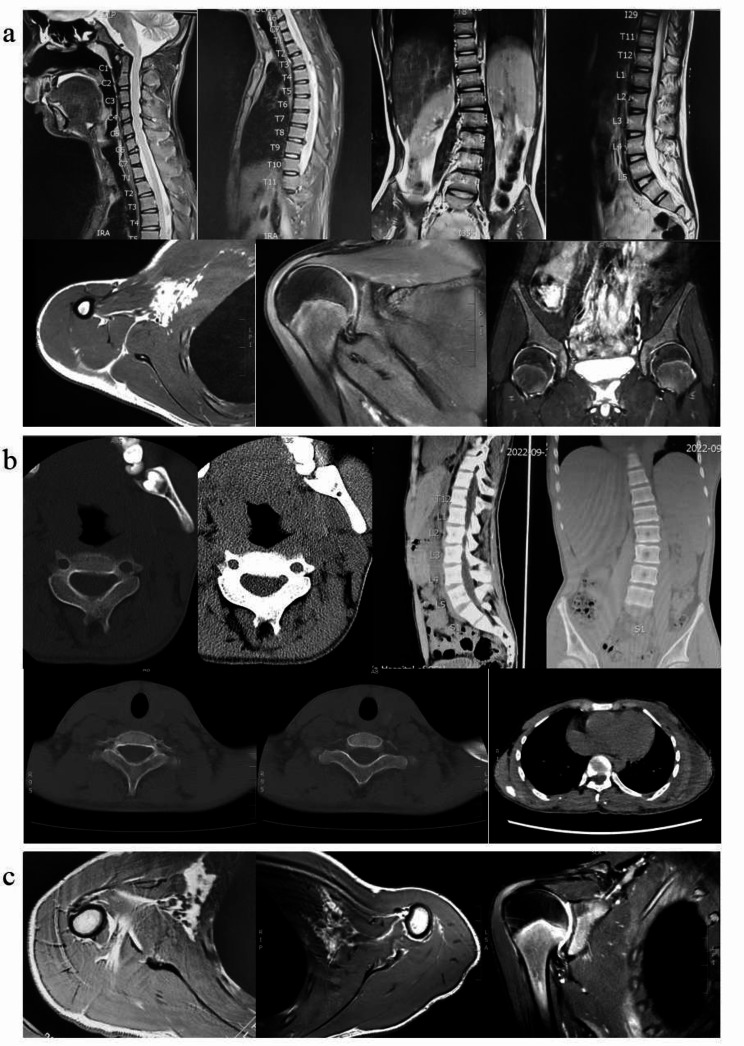
Imaging studies. (**a**) MRI of the first admission; (**b**) CT of the second admission; (**c**) MRI of the second admission

The X-ray showed the femoral heads and humeral heads were abnormally enlarged bilaterally. The spine curves to the left at the seventh thoracic vertebra and to the right at the third lumbar vertebra. The lumbar ribs can be seen on the right of the first lumbar vertebra. It also suggested scoliosis and lumbarization of the first sacral vertebra. The magnetic resonance imaging (MRI) (Fig. 2a) revealed soft tissue and muscle edema, bone marrow edema at the femoral head-neck junction, and superficial myofascitis in the neck, back and waist. There was extensive edema of the right pectoralis major, subscapularis, deltoid, long head of biceps brachii and surrounding soft tissues. Multiple enlarged lymph nodes were found in the right neck and axilla. Electromyography found a large number of narrow-duration, low-amplitude MUP at bilateral T9 paraspinal muscles, and the possibility of myogenic damage could not be excluded. Whole body bone scan showed scoliosis and there were metabolically active spots in thoracic 11/12 vertebrae, which could be degeneration. Apparently, the patient at this time presented with muscle and soft tissue edema and did not have the typical imaging findings of extraskeletal ossification in FOP. Vitamin D (800IU, qd. po.), Calcium Carbonate and Vitamin D3 Tablets (600 mg, qd. po.), and neurotrophic support were administered to the patient. Rehabilitative care such as magnetic-heat therapy and microwave therapy could be an important adjunct to drug treatments. The pain was relieved and the right shoulder movement range was expanded to 85° elevation after treatment. After vitamin D supplementation, the 25-hydroxyvitamin D level was 322 nmol/L (reference range, 75–250 nmol/L).

**Table 1:** Laboratory evaluation

	result	reference range	unit
White blood cell	5.82	3.50–9.50	10^9/L
Hemoglobin	130	130–175	g/L
Neutrophilic granulocyte	3.01	1.80–6.30	10^9/L
Platelet	261	125–350	10^9/L
C-reactive protein	2.15	0–6	mg/L
Erythrocyte Sedimentation Rate	3	0–15	mm/h
Alanine aminotransferase	4.4	9.0–50.0	U/L
Aspartate aminotransferase	16.5	15.0–40.0	U/L
Albumin	38.7	40–55	g/L
Blood Urea Nitrogen	3.35	2.90–7.14	mmol/L
Creatinine	51.6	44.0-133.0	umol/L
Uric Acid	327.9	208.0-428.0	umol/L
Sodium	138.5	137.0-147.0	mmol/L
Potassium	4.34	3.50–5.30	mmol/L
Calcium	2.27	2.11–2.52	mmol/L
Human leukocyte antigen-B27	negative(-)	negative(-)	-
Immune globulin A	< 18	< 18	u/ml
Immune globulin M	< 18	< 18	u/ml
Immune globulin G	< 18	< 18	u/ml
Antinuclear antibody(1:80)	negative(-)	negative(-)	-
Anti-cyclic citrullinated peptide antibody	1.51	0.00–20.00	U
Extractable nuclear antigen	negative(-)	negative(-)	-

**Table 2:** Bone turnover markers, BTMs

	result	reference range for males
Bone glutamyl protein	219 ng/ml	14–46 ng/ml
Total procollagen type 1 amino-terminal propeptide	> 1200 ng/ml	9.06–76.24 ng/ml
β-Cross	2742 pg/ml	47–783 pg/ml

**Table 3:** Quality control data of whole exome sequencing

	Sequencing Quality Control Data
Gene Count	20,324
Coding region Count	192,116
Base Count (bp)	37,012,868
Average depth	215 ± 101
Coverage ( > = 10x)	98.5%
Coverage ( > = 20x)	96.6%

**Fig. 3:**
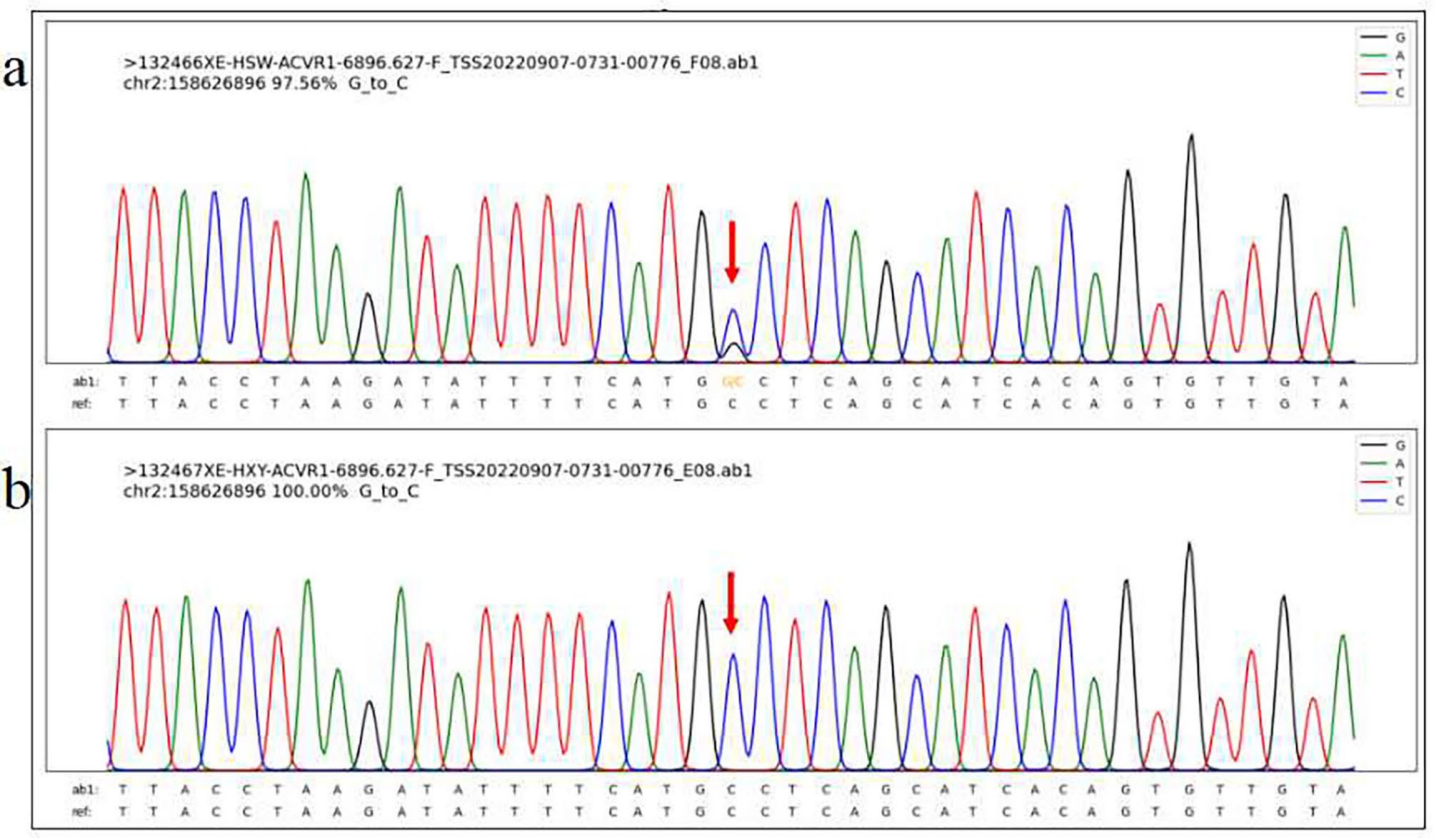
Genetic test. (**a**)Variation correlation map of the patient; (**b**)Variation correlation map of the patient’s father

**Fig. 4:**
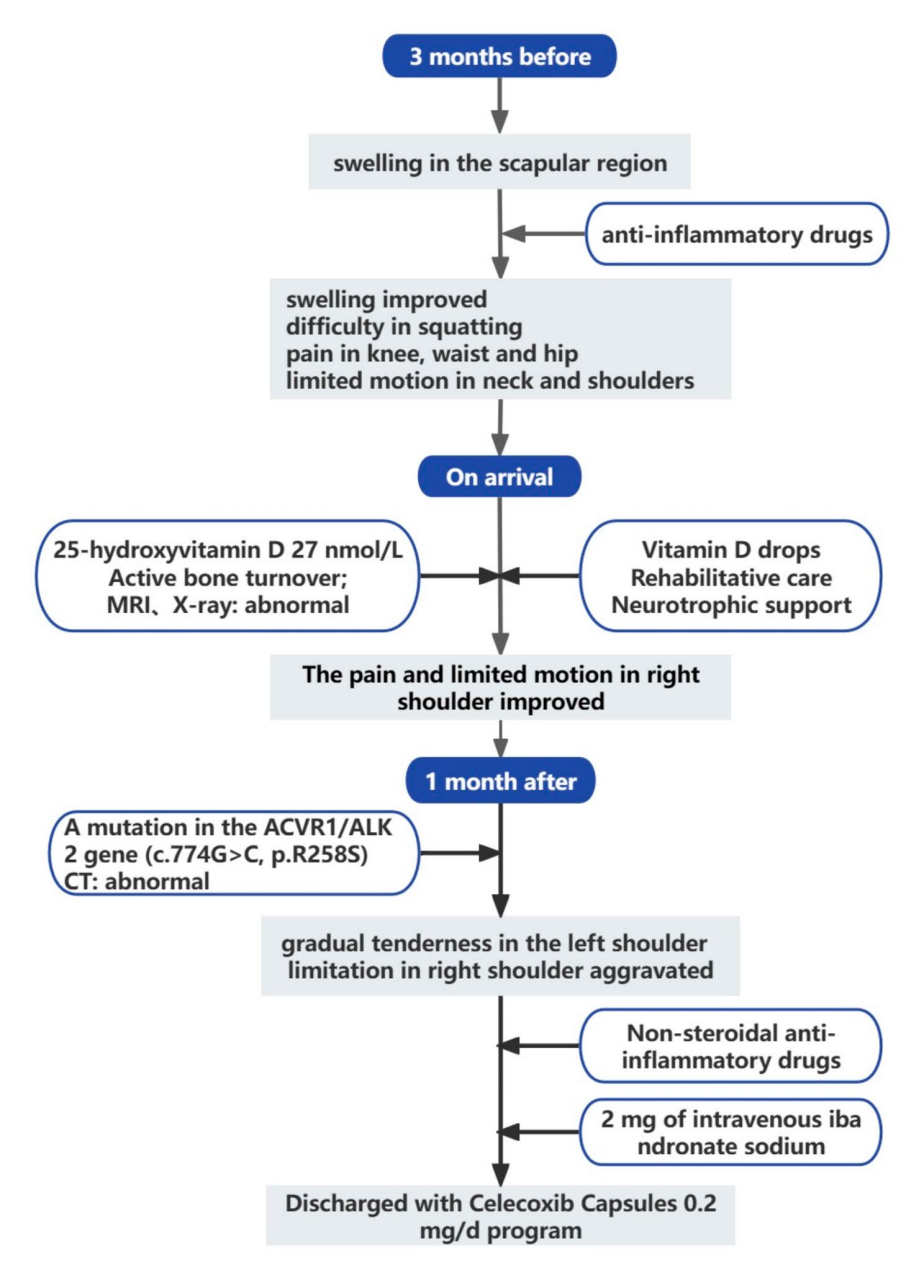
Flowchart of disease progression for the patient

However, the patient has gradual tenderness in the left shoulder, The elevation range of the left shoulder was restricted to 110° after 1 month. The patient had no tenderness in the right shoulder joint and lower back. But both knee joints still had tenderness and difficulty in squatting. The subsequent genetic test identified a mutation in the *ACVR1/ALK2* gene (c.774G > C, p.R258S) (Fig. 3a). Although the patient’s father did not present with clinical features similar to those of the patient, such as pain, swelling, and limited mobility, we still refined his genetic testing (Fig. 3b). Variation correlation map of the patient’s father indicated that he did not have the mutation in the *ACVR1/ALK2* gene, thus demonstrating the de novo origin of the mutation. On the basis of the result of genetic test, the patient was eventually diagnosed with FOP (the disease progression is shown in Fig. 4). Computed tomography (CT) (Fig. 2b) found fusion of posterior elements of the cervical spine and scoliosis, and multiple bony structures were formed in the posterior of the thoracolumbar spine, the bilateral subscapular angle area, the muscle of right chest wall and the left gluteus medius. Progressive heterotopic ossification, as the typical clinical phenotypes of FOP, also confirms the diagnosis of FOP. A re-examination of MRI (Fig. 2c) showed that the extensive edema in the original right pectoralis major muscle, subscapularis muscle, deltoid muscle, long head of biceps brachii muscle and the surrounding soft tissue were reduced and relieved, and the enlarged lymph nodes in the right axilla were reduced. There was extensive soft tissue edema in the left pectoralis major, subscapularis, bilateral chest and back muscles, intermuscular space, and axilla, and slight enlargement of the left axillary lymph nodes. This is consistent with the patient’s reduced pain in the right shoulder and progressive tenderness in the left shoulder.

The intravenous injection of 2 mg of ibandronate sodium was given to the patient. The patient was discharged with Celecoxib Capsules 0.2 mg/d. The drug dosage would be gradually suspended after 2 weeks when the patient’s joints pain gets remission. At the last follow-up, he had a lower bone turnover rate than before with the β-Cross level of 1351 pg/ml (reference range, 47–783 pg/ml) and the total procollagen type 1 amino-terminal propeptide level of 718.40 ng/ml (reference range, 9.06–76.24 ng/ml).

## Discussion

This patient was diagnosed with FOP, which is an unusual disabling autosomal dominant genetic disease. A study by Arun-Kumar Kaliya-Perumal et al. indicated the typical clinical phenotypes of FOP, including the typical congenital deformities of the great toe and progressive HO with other atypical signs of FOP such as spinal malformations, tibial osteochondromas, thumb malformations, broad femoral neck, sparse hair and eyebrows, late-occurred HO and uncharacteristic great toe deformities [20]. And these atypical signs are found in patients with atypical mutations. On the basis of the data from the International FOP Association (IFOPA), the average age for patients to have the initial symptoms of FOP is 5.4 years, with an average age of 7.5 years for final diagnosed FOP [21]. Patients with FOP succumb early as thoracic insufficiency syndrome, trauma, or infectious diseases [22]. Due to the complexity of FOP, the diagnosis remains a challenge for endocrinologists. Sharing relevant information about FOP researches can help to improve accessibility of care and address these challenges [23].

In 2006, it was determined that the causative variant of FOP was *ACVR1/ALK2* gene [24]. In the classic form of FOP, the *ACVR1* gene c.617G > A leads to a missense mutation of codon 206, which results in replacement of arginine with histidine at position 206 (p.R206H) of the ACVR1 protein. Atypical missense mutations have also been identified in some FOP patients, and in our case, the *ACVR1* gene c.774G > C causes a substitution mutation from arginine to serine at position 258 (p.R258S) of the ACVR1 protein. These substitutions alter the properties of the receptor causing the activation of the receptor without an exogenous ligand and phosphorylation of Smad proteins. However, Alessi Wolken DM et al. suggested that the mutated receptor can incorrectly regard activin as a BMP and elicit phosphorylation, leading to activation of the endochondral ossification transcription programme [25]. Another research indicated that elevated basal phosphorylated SMAD1/5/9 activity, tissue damage and inflammation can induce BMP, resulting in a skeletogenic signal in the absence of ligands [26]. Subsequently, mesenchymal cells at the inflammation site differentiate, leading to transformation of the fibroblast-rich tissue into cartilage matrix, which is ultimately superseded by bone via an endochondral process. So, during the early stages of the disease, there was localized edema and inflammatory cell infiltration in muscles, tendons and myofasciae. Then, cartilages replace early fibroplasia and most of them metamorphose into mature heterotopic bones, and we can find calcification of muscle and soft tissue by imaging examinations.

In the previous literature, FOP has been diagnosed only after extensive heterotopic ossification and dysfunction, which has caused the patients to lose function and miss the optimal treatment time window. However, our patient was clinically and genetically diagnosed at the stage of joint swelling, which is particularly infrequent in clinical practice. Early detection of FOP can effectively avoid iatrogenic damage caused by inappropriate and unnecessary testing. Our case may help clinicians diagnose patients during the early periods of illness. The young male patient in our case presented with swelling in the scapular region at the onset of the disease. And the swelling lesion resolved after anti-inflammatory treatment. However, in subsequent days, pain and limited motion in several parts of the body gradually appeared. Imaging examination found muscle and soft tissue swelling, as well as scoliosis and lumbarization of the first sacral vertebra. In addition, the patient had slight abnormalities of the great toe. Our patient exhibited many FOP-related features such as recurrent painful swelling and tenderness of soft tissues, normal inflammatory indicators, joint deformity and limited mobility, which is highly suggestive of FOP. Although the clinical presentation and course of our patient closely resemble those of FOP, distinguishing it diagnostically from other diseases could have significant implications (Table 4). Patients with osteogenesis imperfecta (OI) can also present with skeletal deformities, but our patient did not have the characteristic bone fragility and recurrent fractures of OI. OI results from changes in type I collagen [27]. And patients with OI may also present blue-gray sclera, odontic deformities, joint overmobility, hearing damage and muscle weakness [28], which are also inconsistent with our patient. In addition, spondyloepiphyseal dysplasia congenita (SEDC) can also cause scoliosis and joint deformities. However, it is distinguished by short trunk, aberrant epiphysis, and flattened vertebral body, and the cause of SEDC is identified as the mutation in the *COL2A1* gene [29]. Besides, Mucopolysaccharidosis (MPS) can also involve bones, cartilage, ligaments, tendons, articular capsules, and other soft tissues around the joints. But skeletal changes observed in MPS are the Madelung’s deformity, short and incrassate clavicles, and oar-shaped ribs, and the bullet-shaped metacarpals [30], which is not consistent with our patient’s symptoms. An elevated glycosaminoglycan concentration in urine can help identify MPS, but definitive diagnosis requires enzyme activity assays [31].

**Table 4:** The differential diagnosis of other genetic bone diseases

	Clinical features	Diagnostic markers	Genetic differences
Fibrodysplasia ossificans progressiva	Congenital malformation of the great toe, progressive heterotopic ossification	Heterotopic ossification and genetic testing	Mutation in *ACVR1/ALK2* gene
Osteogenesis imperfecta	Bone fragility, recurrent fractures, blue-gray sclera and odontic deformities	Bone fragility and genetic testing	Mutation in *COL1A1* or *COL1A2* gene
Spondyloepiphyseal dysplasia congenita	Short trunk, aberrant epiphysis, and flattened vertebral body	Genetic testing	Mutation in the *COL2A1* gene
Mucopolysaccharidosis	The Madelung’s deformity, short and incrassate clavicles, oar-shaped ribs, bullet shape metacarpals	Glycosaminoglycan concentration in urine, enzyme activity assays	Mutation in the *IDUA* gene

Then, to differentiate FOP from other diseases, we suggested he performed the sequencing of the *ACVR1/ALK2* gene from a peripheral blood sample. By the presence of the mutation c.774G > C, p.R258S (HGVS: NC_000002.11:g.158626896 C > G) in the *ACVR1/ALK2* gene, the clinical suspicion is confirmed. Then we reviewed the imaging examination and found typical heterotopic ossification of FOP in the back and scapular region, the muscle of right chest wall and the left gluteus medius. The clinical manifestations were consistent with the genetic diagnosis. Finally, the patient was diagnosed with FOP and effectively treated with ibandronate sodium and non-steroidal anti-inflammatory drugs. Previous studies have shown that the R258 residue is highly conserved among species [32–33]. The study of Bocciardi R et al. described a patient carrying this new *ACVR1* c.774G > C, p.R258S mutation who had deformation of the great toe, even though this seemed quite slight, which was consistent with our case findings. And another patient in the study is heterozygous for a c.44 C > G, p.A15G substitution in exon 1 with the novel *ACVR1* mutation c.774G > C, p.R258S. She had painful swelling in the cervical vertebral region and progressive heterotopic ossification whose striking peculiarity was the absence of great toe deformation [32]. The study of Nakahara Y found that the patient with c.774G > T, p.R258S who also has the same amino-acid change (p.Arg258Ser) has body movement difficulties and heterotopic ossification around the paraspinal muscles and bilateral shoulder and hip joints without great toe malformation and spinal deformity. So phenotypic differences may exist between c.774G > T, p.R258S and c.774G > C, p.R258S, which is whether there is the great toe malformation [33]. However, there is too few data on these mutations to prove the hypothesis of the genotype–phenotype correlation, and our case can help add relevant information.

Therapy of FOP is focused on treating with NSAID and large dose corticosteroids upon presentation. Celecoxib, an NSAID, has shown efficacy in reducing HO formation [34]. However, indomethacin, another NSAID, did not block FOP [35]. So selection of effective NSAIDs for FOP may be helpful. Bisphosphonates like pamidronate and zoledronate have been used empirically to treat the symptomatic FOP flare-ups [36], and there was significant amelioration in some of cases. The study of Haviv R et al. indicated IL-1 inhibitors may be a latent strategy for reducing flare activity of FOP [37]. Furthermore, rehabilitative care can also ease FOP-associated pain. The discovery of mutations in the *ACVR1/ALK2* as the etiology of FOP provides emerging targeted therapies and certain such therapeutic strategies are in development which hold great promise for managing FOP [38,^39^]. These include ALK2 inhibitors, such as Saracatinib, which efficacy against HO in preclinical models [40]. Saracatinib can efficiently inhibit ALK2 signaling in a selective manner, thereby inhibiting chondrogenic differentiation. In addition, Garetosmab [41], an activin A-blocking monoclonal antibody, can block the ability of activin A to activate FOP-mutant *ACVR1*. The phase 2 trial demonstrated that Garetosmab can treat pre-existing HO lesions and prevent new HO from taking shape. And the phase 3 trial and researches in Children of Garetosmab are expected. Another strategy of treating FOP was identified for rapamycin (mTOR) inhibitors or hypoxia-inducible factor 1-Alpha (HIF-1α) inhibitors which reduced the production of HO in FOP mouse models [42,43]. Rapamycin affects hypoxic signaling and inflammatory signaling in chondrogenesis by influencing mTOR signaling pathway [44]. Besides, retinoic acid receptor gamma agonists including Palovarotene offer targets of therapeutic intervention by activating the retinoid signaling pathway and down-regulating the BMP signaling pathway. The phase 3 trial [45] showed Palovarotene prevent the premier and decisive chondrogenic stage resulting in HO onset and progression, but there is high risk of epiphyseal disorder. Another therapeutic target under investigation for FOP treatment is transforming growth factor beta (TGF-β) [46]. Elevated levels of active TGF-β induce and promote FOP by triggering Activin A production. So the inhibitory effect of TGF-β activity by injecting TGF-β neutralizing antibodies may attenuate HO progression in Activin A-mediated FOP. Moreover, phosphoinositide 3-kinases (PI3K)-inhibitors show the potent therapeutic effect on HO by influencing both the chondrogenic and osteogenic steps of heterotopic ossification [47].

A bibliometric analysis of the 20-year period indicated that the genetic aetiology, pathogenic mechanism, and therapy of FOP became research hotspots [48]. Palovarotene was approved firstly by the Food and Drug Administration to treat FOP [49], and other four drugs are in trials, including BLU-782 (IPN60130), a ALK2 R206H inhibitor, which could prevent HO in FOP [50]. The latest research demonstrated that suppression of cell cilia could be a treatment strategy for FOP [51]. Furthermore, targeting senescent cells [52], immune modulators [53], Ihh or Yap inhibitors [54], Matrix metalloproteinase-9 inhibition [55], NF-κB [56], oxidative phosphorylation [57] and AAV-based gene therapy [58] may also be the key targets for treatment at different stages of FOP. Early diagnosis of FOP is important for early prevention and treatment, which can help to avoid injury or iatrogenic harm and ameliorate the pain of flare-ups. Diagnosis and therapy of FOP lag behind the detection of HO, so it is significant to learn from developments made in the research of traumatic HO and genetically mediated HO [59]. The correlation between genotype and phenotype remains indeterminate, necessitating further studies to augment data on various mutations for enhanced diagnosis, comprehension, prevention, and treatment of FOP.

## Conclusions

FOP, a rare genetic disease, has complicated and volatile clinical pictures, which makes the diagnosis troublesome. And delayed or insufficient diagnosis will touch off flare-up and HO. However, the recognition of hallmark signs and utilization of imaging studies like CT and MRI can greatly aid in early suspicion of FOP. Then, alert physicians can confirm the diagnosis of FOP through genetic testing. This case highlights the importance of the differential diagnosis of FOP by endocrinologists, and helps accumulating data on novel mutations of FOP.

## Data Availability

The data that support the findings of this study are available from The Second Xiangya Hospital of Central South University but restrictions apply to the availability of these data, which were used under license for the current study, and so are not publicly available. Data are however available from the authors upon reasonable request and with permission of The Second Xiangya Hospital of Central South University.

## Abbreviations

ACVR1/ALK2: Activin A receptor type I/activin-like kinase 2
BMP: Bone morphogenetic protein
BTMs: Bone turnover markers
CT: Computed tomography
FOP: Fibrodysplasia ossificans progressiva
GS: Glycine-serine-rich
HIF-1α: Hypoxia-inducible factor 1-Alpha
HO: Heterotopic ossification
IFOPA: International FOP Association
MPS: Mucopolysaccharidosis
MRI: Magnetic resonance imaging
Mtor: Mammalian target of rapamycin
NSAID: Non-steroidal anti-inflammatory drugs
OI: Osteogenesis imperfecta
PI3K: Phosphoinositide 3-kinases
PK: Protein kinase
SEDC: Spondyloepiphyseal dysplasia congenita
SMAD: Small-mothers-against decapentaplegic
TGF-β: Transforming growth factor beta
TM: Transmembrane
